# Proton magnetic resonance spectroscopy: clinical applications in patients with brain lesions

**DOI:** 10.1590/S1516-31802003000600008

**Published:** 2003-11-06

**Authors:** Sérgio Luiz Ramin, Waldir Antonio Tognola, Antonio Ronaldo Spotti

**Keywords:** Proton, Spectroscopy, Magnetic resonance, Brain, Espectroscopia de prótons, Ressonância magnética, Encéfalo

## Abstract

**CONTEXT:**

Proton spectroscopy has been recognized as a safe and noninvasive diagnostic method that, coupled with magnetic resonance imaging techniques, allows for the correlation of anatomical and physiological changes in the metabolic and biochemical processes occurring within previously-determined volumes in the brain. There are two methods of proton magnetic resonance spectroscopy: single voxel and chemical shift imaging

**OBJECTIVE:**

The present work focused on the clinical applications of proton magnetic resonance spectroscopy in patients with brain lesions.

**CONCLUSIONS:**

In vivo proton spectroscopy allows the detection of certain metabolites in brain tissue, such as N-acetyl aspartate, creatine, choline, myoinositol, amino acids and lipids, among others. N-acetyl aspartate is a neuronal marker and, as such, its concentration will decrease in the presence of aggression to the brain. Choline increase is the main indicator of neoplastic diseases. Myoinositol is raised in patients with Alzheimer's disease. Amino acids are encountered in brain abscesses. The presence of lipids is related to necrotic processes.

## RESUMO

**CONTEXTO:**

A espectroscopia de prótons é reconhecidamente um método não invasivo que, quando associada à imagem por ressonância magnética, possibilita a correlação de alterações metabólicas e bioquímicas com mudanças fisiológicas e anatômicas dentro de um volume previamente determinado no encéfalo. Existem dois métodos de espectroscopia de prótons por ressonância magnética: volume único de interesse (*single voxel*) e imagem espectroscópica (*chemical shift imaging*).

**OBJETIVO:**

Este trabalho discute as aplicações clínicas da espectroscopia de prótons por ressonância magnética em pacientes com lesões encefálicas.

**CONCLUSÕES:**

A espectroscopia de próton por ressonância magnética *in vivo* permite detectar alguns metabólitos existentes no tecido encefálico, como N-acetil aspartato, creatina, colina, mio-inositol, aminoácidos, lipídios, entre outros. O N-acetil aspartato é um marcador neuronal cuja concentração diminui quando há lesão encefálica. O aumento na concentração de colina é o principal indicador de doenças neoplásicas. A quantidade de mio-inositol apresenta-se aumentada em casos de doença de Alzheimer. Aminoácidos estão presentes em abscessos encefálicos. A presença de lipídios está relacionada à necrose tecidual.

## INTRODUCTION

The basic principles of magnetic resonance have been known since the 1940s, but due to technical difficulties the first imaging of the human body via magnetic resonance was only achieved at the beginning of the 1980s. In contrast, between the development of the principles of x-rays and the first images produced by that method there was a time interval of only four months.^1^

Magnetic resonance imaging is an excellent method for anatomical and structural diagnosis of the brain, but it does not provide functional or metabolic information. At the beginning of the 1990s, one of the options for assessing the metabolic and functional activity of the brain was positron emission tomography or functional magnetic resonance (diffusion, perfusion and spectroscopy),^2,3^ which was used mainly in research institutes. The equipment necessary for this purpose was expensive and inaccessible for most medical centers of the world and still is, in the case of positron emission tomography.

In the mid-1990s, however, the development of computer software for spectroscopy coupled to the previously existing magnetic resonance equipment contributed towards reducing prices. For this reason, the clinical use of spectroscopy using *in vivo* magnetic resonance has become routine in many hospitals.^4^

### Magnetic resonance spectroscopy

Magnetic resonance spectroscopy is used to detect the metabolic and biochemical profile of brain areas.^4^ Several chemical elements can be used to obtain magnetic resonance spectroscopy such as phosphorus,^5-7^ carbon^8,9^ and hydrogen.^10^

The first *in vivo* magnetic resonance spectroscopy was performed at the beginning of the 1980s and was done using resonance of the phosphorus nucleus (^31^P), thereby revealing the energy metabolism of the tissue cells studied.^7^ However, this method had the disadvantages of low magnetic sensitivity, low concentration of ^31^P atoms and also inadequate spatial resolution in focal brain lesions with small and mild dimensions,^7^ and it has been replaced by proton (hydrogen) spectroscopy, especially in brain studies.^10^

Proton (^1^H) resonance is nowadays the method most frequently used in neurospectroscopy, because hydrogen is the most abundant atom in the human body and its nucleus emits the most intense radio-frequency signal, when in an external magnetic field, in relation to other nuclei.^10^ Moreover, proton magnetic resonance spectroscopy is more quickly accomplished and easily interpreted.

### *In vivo* proton magnetic resonance spectroscopy

In 1989, Frahm et al.^11,12^ published the first reports of *in vivo* proton magnetic resonance spectroscopy, describing the methodology used in the detection and measurement of metabolite concentrations in the human brain.

Proton spectroscopy has been recognized as a noninvasive method, approved since 1996 by the Food and Drug Administration (FDA). Coupled with magnetic resonance imaging techniques, it allows for the correlation of anatomical and physiological changes in the metabolic and biochemical processes occurring within previously-determined volumes in the brain.^13^

Proton magnetic resonance spectroscopy of the brain is useful whenever biochemical or metabolic assessment may be necessary, such as in differential diagnosis of focal brain lesions (neoplastic and non-neoplastic diseases);^14-20^ brain lesions in patients with acquired immunodeficiency syndrome;^21-23^ diagnosis of dementia^24-26^ and other degenerative diseases;^27^ follow-up radiation therapy for patients with brain neoplasms;^28-30^ demyelinating diseases such as multiple sclerosis^31-33^ and leukodystrophy;^6,34^ diagnosis and prognosis of brain ischemic^35,36^ and traumatic lesions;^37-42^ assessment of epilepsy;^43-45^ biochemical alterations in hepatic encephalopathies;^46,47^ and neuropediatric affections such as brain tumors, inborn errors of metabolism and hypoxic encephalopathy.^48-50^

Magnetic resonance examinations, including spectroscopy, are absolutely contraindicated in patients with heart pacemakers or other electronic appliances implanted in the body, and those with steel clips in brain aneurysms. Individuals with claustrophobia and children need sedation.^51^

### Techniques

There are two methods of proton magnetic resonance spectroscopy: single voxel and multivoxel, with or without spectroscopic imaging. Single voxel proton magnetic resonance spectroscopy provides a rapid biochemical profile of a localized volume within a region of interest that may be determined, especially in brain studies.^4,16,52,53^ Spectroscopic imaging provides biochemical information about multiple, small and contiguous volumes focalized on a particular region of interest that may allow the mapping of metabolic tissue distribution. By using this method, the data obtained may be manipulated by computer and superimposed on the image of an abnormality, thereby illustrating the distribution of such metabolites within that area.^54-56^

The two localization methods commonly used in clinical proton magnetic resonance spectroscopy are PRESS (point-resolved spectroscopy) and STEAM (stimulated echo acquisition mode). Both methods stimulate protons within the volume of interest with minimal stimulation outside of this volume.^11,13,36,52,57-61^

Another essential variable in the acquisition of proton magnetic resonance spectroscopy is the choice of echo time. With short echo times (less than 30 milliseconds), the magnetic resonance spectrum detects larger numbers of metabolites, but it is more likely that peak superimposition will occur, thereby causing difficulty in spectroscopic curve interpretation. Short echo times are indicated for the study of metabolic and diffuse diseases.^52^ By using long echo times (more than 135 milliseconds), smaller numbers of metabolites are detected, but with better definition of peaks, thereby facilitating graphic analysis. Long echo times are more used in focal brain lesions.^62^

### Metabolites: localization and importance

*In vivo* proton magnetic resonance spectroscopy allows the presence of certain metabolites in brain tissue to be detected if the minimum concentrations are between 0.5 and 1.0 mM.6 Some of these present clinical importance,^25,48,49,63-72^ such as:

1. N-acetyl aspartate (Naa) – this is a neuronal marker that is present in neuron bodies and axons, and indicates their density and viability. Its production takes place in the mitochondria of brain tissue. Because of these factors, the Naa peak in proton spectroscopy will be decreased whenever there is neuron loss, such as in glioma, ischemia and degenerative diseases. Naa presence resonates at 2.02 parts per million (ppm)
2. Creatine (Cr) – this is a marker of the aerobic energy metabolism of the brain cells, and is present in larger concentrations in the gray matter than in the white. The creatine peak is practically constant and may be used as a control value in relation to other metabolites. Creatine phosphate also contributes to the Cr peak. Occasionally, a reduction in the Cr peak occurs in brain tumors, mainly in metastases. The peak for Cr is seen at 3.02 ppm; however, an additional peak for creatine may be visible at 3.94 ppm
3. Choline (Cho) – this is a constituent molecule of the phospholipid metabolism of cell membranes and reflects membrane turnover. Its concentration is slightly greater in white matter than in gray matter. Increased choline indicates greater membrane synthesis and cell proliferation. Its concentration is normally greatly increased in cases of brain neoplastic expansible processes. Phosphocholine and glycerophosphocholine also contribute to the representation of the Cho peak, which occurs at 3.2 ppm
4. Lactate – this is not commonly detected in proton spectroscopy of brain tissue. Its presence indicates a pathological condition with regard to the final products of anaerobic metabolism. Lactate can be identified in cysts, hypoxic/ischemic tissues and some neoplasms. It is visible as an inverted double peak on the spectroscopic curve (echo time of 136 ms) at 1.33 ppm
5. Lipids – these metabolites are usually not detected by proton magnetic resonance spectroscopy, either. In pathological situations in which necrosis occurs, such as in malignant neoplasms and inflammatory/infectious processes, there is an accentuated lipid peak, signifying cell membrane degradation. This peak is located at 0.9 and 1.3 ppm
6. Myoinositol – this is considered to be a glial function marker, and it is an important osmotic agent regulator for cell volume. It generally presents reduction in hepatic encephalopathy and elevation in Alzheimer's disease. The myoinositol peak occurs at 3.56 ppm.

In pathological cases, other metabolites that can be detected via proton magnetic resonance spectroscopy include the following amino acids: a) alanine, as an inverted double peak in meningiomas and brain abscesses (at 1.48 ppm with an echo time of 136 ms); b) acetates and succinates in abscesses and neurocysticercosis (at 1.92 and 2.4 ppm, respectively); and c) cytosols in abscesses (at 0.9 ppm).

### Interpretation of the spectroscopic curve

**Figure 1:**
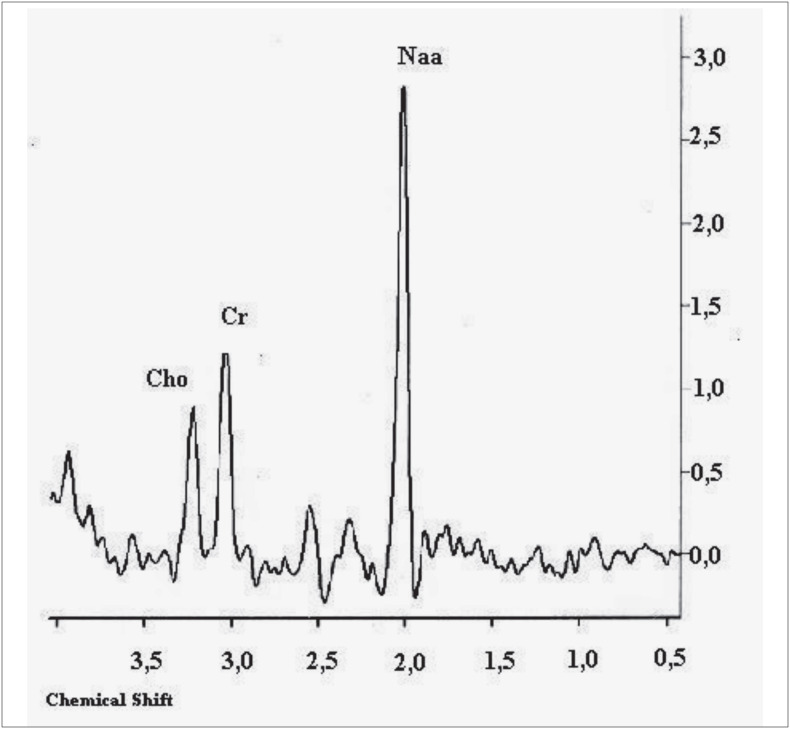
Normal brain curve from proton magnetic resonance spectroscopy of the brain, showing peaks of the metabolites Nacetyl aspartate (Naa), creatine (Cr) and choline (Cho), with echo time of 136 milliseconds.

The spectrum represents radiofrequency signals emitted from the proton nuclei of the different metabolites into the region of interest. Specific metabolites always appear at the same frequencies, expressed as parts per million, and are represented on the horizontal axis of the graph. The vertical axis shows the heights of the metabolite peaks, represented on an arbitrary intensity scale. Figure 1 shows proton spectroscopic curves for the magnetic resonance of normal brain tissue, with the Nacetyl aspartate, creatine and choline peaks.

The study of proton magnetic resonance spectroscopy can be either qualitative or quantitative. Qualitative study results in graphs of metabolites from a given region of the brain. Quantitative study measures the real concentration of metabolites, which is proportional to the area under the peak, or the relationship of their rates with each other, through height measurement of the peaks on the graph.

### Main clinical applications

#### Brain tumors

**Figure 2:**
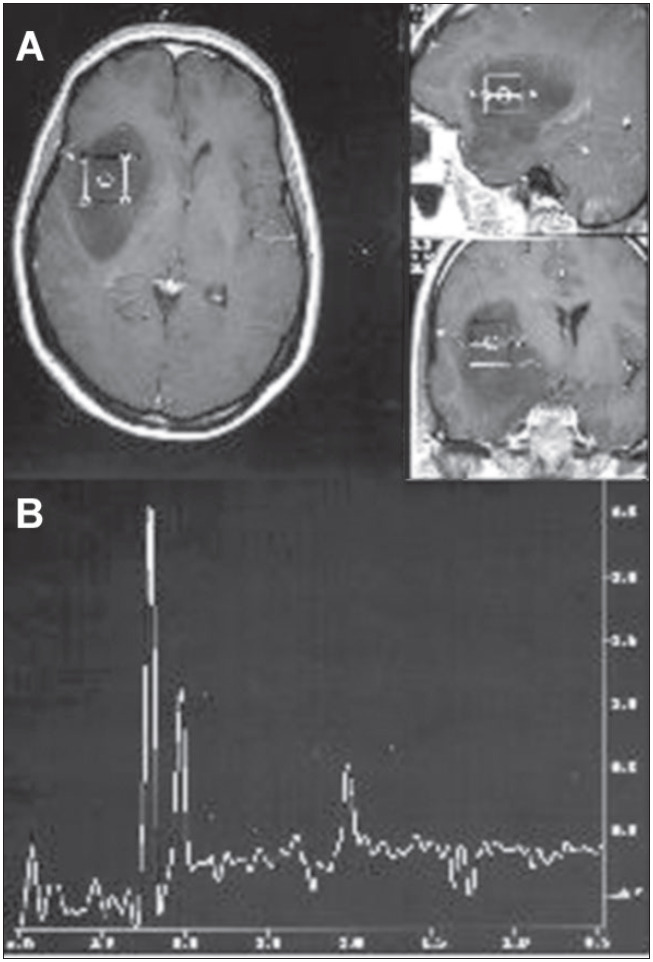
A-Magnetic resonance imaging in the axial, sagittal and coronal planes of the brain (T_1_-weighted), showing hypointense frontoparietal lesion without postgadolinium enhancement, in voxel position, typical of low-grade astrocytoma. B-Spectroscopic curve from proton magnetic resonance with echo time of 136 milliseconds. Note inverted double peak of lactate (1.33 ppm), decrease in the N-acetyl aspartate peak (2.02 ppm) and pronounced increase in the choline peak (3.2 ppm).

Proton magnetic resonance spectroscopy is a very sensitive method for detecting brain tumors. Decreased intensity of the N-acetyl aspartate peak and increased choline occur in gliomas (Figure 2).^25^ Lactate peaks may be found in such tumors, independent of their malignancy grade,^33,53^ thereby indicating hypoxia. There is controversy regarding the capacity of proton spectroscopy to distinguish between different histological grades of gliomas; however, the detection of lipids is typical of multiform glioblastoma, i.e. tissue necrosis.^14^ When the image obtained by magnetic resonance does not succeed in differentiating glioma from infection, the proton magnetic resonance spectroscopy is useful, because in neoplastic processes there is a remarkable increase in the choline peak.^73,74^ Moreover, this method is important in monitoring responses to the treatment of gliomas.^75^

**Figure 3:**
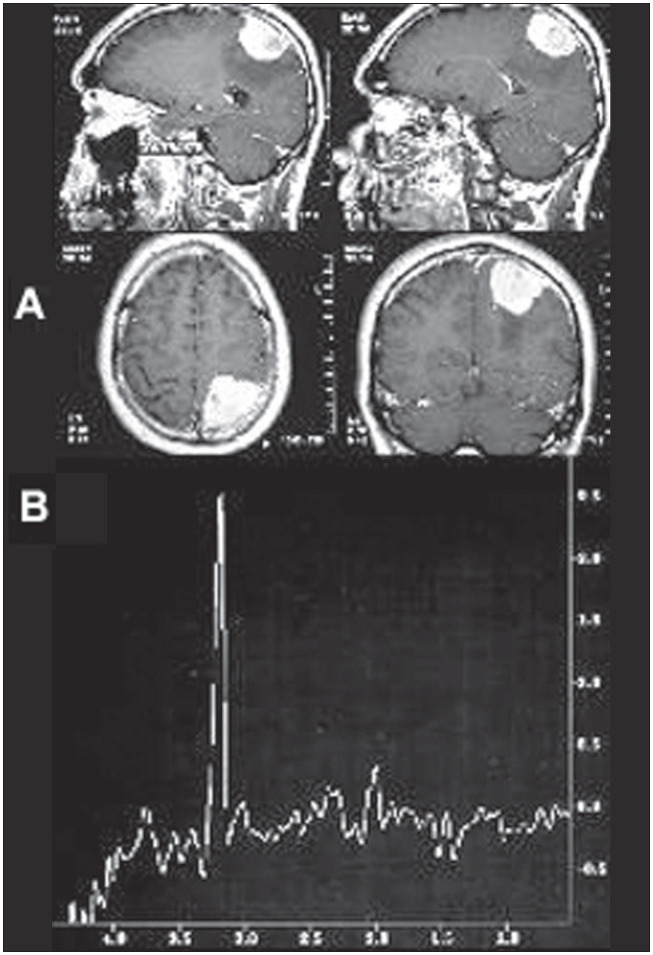
A- Magnetic resonance imaging of the brain in the axial, sagittal and coronal planes (T_1_-weighted), showing parietal lesion with homogeneous postgadolinium enhancement, typical of meningioma. B- Spectroscopic curve from proton magnetic resonance with echo time of 136 milliseconds, demonstrating inverted double peak of alanine (1.48 ppm), accentuated decrease in the N-acetyl aspartate peak (2.02 ppm) and pronounced increase in the choline peak (3.2 ppm).

Although the diagnosis of meningioma is easily done by means of magnetic resonance imaging, proton magnetic resonance spectroscopy may be useful in atypical cases. In such tumors there is a pronounced rise in choline levels, associated with absence or considerable reduction of N-acetyl aspartate.^4^ Presence of an alanine peak can confirm the diagnosis (Figure 3).^65^

#### Inflammatory and infectious processes

The main application of proton magnetic resonance spectroscopy in inflammatory and infectious processes is the establishment of differential diagnoses between these processes and brain tumors.

**Figure 4:**
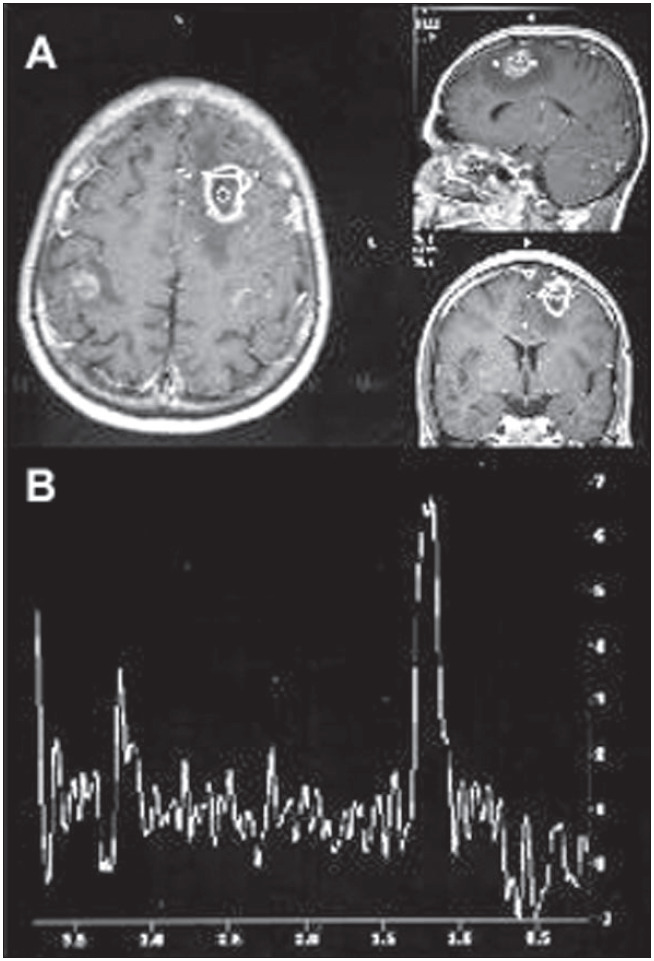
A- Magnetic resonance imaging of the brain in the axial, sagittal and coronal planes (T_1_-weighted), showing frontoparietal ring-like postgadolinium lesion enhancement and voxel position, in patient with toxoplasmosis. B- Spectroscopic curve from proton magnetic resonance with echo time of 136 milliseconds, demonstrating accentuated lipid peak (1.3 ppm), absence of N-acetyl aspartate peak (2.02 ppm) and slight increase in the choline peak (3.2 ppm).

In focal inflammatory processes in patients with acquired immunodeficiency syndrome (aids), such as toxoplasmosis (Figure 4), tuberculosis or cryptococcosis, proton spectroscopy shows a broad lipid peak and occasionally a lactate peak, with a decrease or absence of N-acetyl aspartate and slight increase of choline (Figure 4).^20,22^

In pyogenic abscesses, N-acetyl aspartate, creatine and choline peaks are not detected and the spectroscopic curve presents amino acid peaks, especially succinate, acetate and cytosolic peaks, due to the great quantity of hydrolytic enzymes produced by bacteria, which results in elevated concentrations of proteins and amino acids.^20,41,63^

#### Alzheimer's disease

Proton magnetic resonance spectroscopy shows a reduction of N-acetyl aspartate in the frontoparietal, temporal and hippocampus regions, due to the neuron loss and increase in myoinositol concentration.^25,26^ These results can be found even in light and mild cases of dementia, thus reinforcing the importance of this method for early diagnosis of Alzheimer's disease.^24^ However, there is still controversy regarding the sensitivity of proton magnetic resonance spectroscopy in such cases.

#### Ischemic lesions

**Figure 5:**
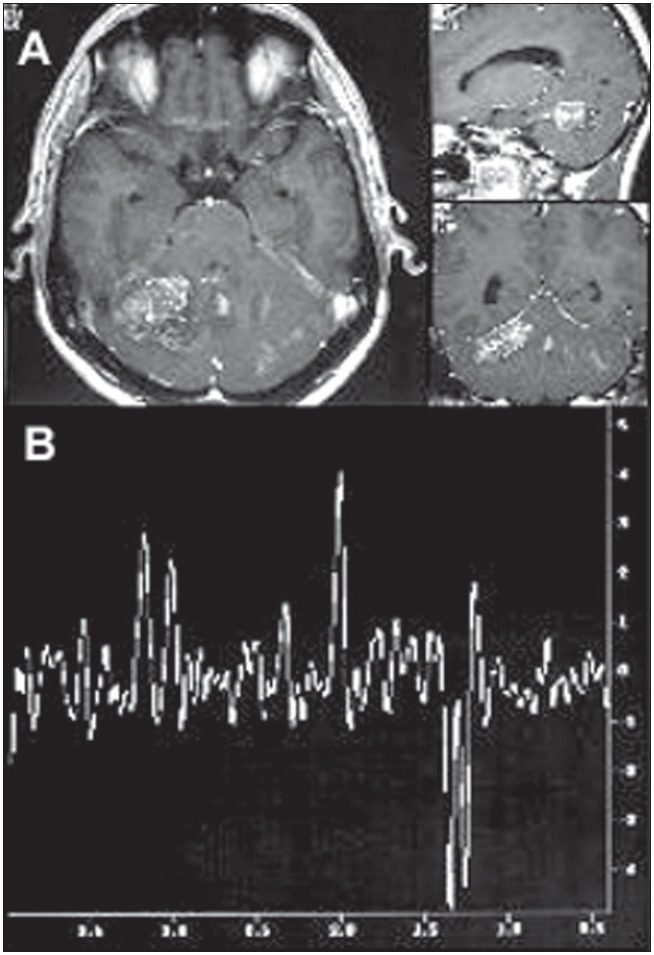
A- Magnetic resonance imaging of the brain in the axial, sagittal and coronal planes (T_1_-weighted), show-ing lesion in the right cerebellar hemisphere with heterogene-ous postgadolinium enhancement and voxel position, in patient with acute ischemia. B- Spectroscopic curve from proton magnetic resonance with echo time of 136 millisec- onds, demonstrating inverted double peak of lactate (1.33 ppm), decrease in N-acetyl aspartate peak (2.02 ppm) and slight increase in the choline peak (3.2 ppm).

The main characteristic of the spectroscopic curve in acute brain ischemia is the early appearance of a lactate peak, decrease of Nacetyl aspartate and slight increase of choline (Figure 5).^33^ It detects lactate within the first minutes of ischemia and, at the subacute phase, its concentration is progressively reduced. The intensity of these peaks in the infarcted area is related to the prognosis.^34^

#### Hepatic encephalopathy

The diagnosis of hepatic encephalopathy in most cases is difficult and, moreover, many patients have a subclinical form of the disease. Proton magnetic resonance spectroscopy shows an elevation of glutamate and glutamine levels (peak between 2.1 and 2.5 ppm) and a reduction in choline and myoinositol levels.^45,46^ These metabolic alterations can be detected before the appearance of lesions through imaging examinations using magnetic resonance.^63^
